# Embryo selection at the cleavage stage using Raman spectroscopy of day 3 culture medium and machine learning: a preliminary study

**DOI:** 10.3389/fendo.2025.1608318

**Published:** 2025-09-15

**Authors:** Fang Cao, Wei Xiong, Xiaohui Lu, Yanjun Luo, Rui Yan, Li Chen, Yufeng Wang, Hanbi Wang, Xiuliang Dai

**Affiliations:** ^1^ The Center for Reproductive Medicine, Changzhou Maternal and Child Health Care Hospital, Changzhou Medical Center, Nanjing Medical University, Changzhou, Jiangsu, China; ^2^ Department of Gynecology Endocrine and Reproductive Center, National Clinical Research Center for Obstetric and Gynecologic Diseases, Beijing, China; ^3^ Department of Obstetrics and Gynecology, Peking Union Medical College Hospital, Peking Union Medical College/Chinese Academy of Medical Sciences, Beijing, China; ^4^ Shanghai D-Band Medical Technology Co., Ltd, Shanghai, China

**Keywords:** embryo selection, extended culture outcomes, spent day 3 culture medium, Raman spectroscopy, machine learning

## Abstract

**Background:**

Blastocyst transfer has been associated with shorter leukocyte telomere length in ART-conceived children, suggesting that extended embryo culture may accelerate aging in offspring. Selecting Day 3 embryos with high developmental potential for transfer could address this issue. The aim of this study is to investigate whether machine learning combined with Raman spectroscopy of spent Day 3 culture medium can serve as a potential method for predicting extended embryo culture outcomes, thereby enabling embryo selection at the cleavage stage.

**Methods**

This prospective study analyzed 172 Day 3 culture medium samples with known extended culture outcomes from 78 couples collected between February 2020 and February 2021. Samples were categorized into three groups based on extended culture outcomes: morphologically good blastocysts (group A), morphologically non-good blastocysts (group B), and clinically non-useful embryos (group C). For each sample, 30–40 Raman spectra were acquired. Machine learning analyses (both unsupervised and supervised) were performed for data visualization and clustering. Eighty percent of the samples from each group were used as training data, while the remaining 20% served as the test set. Twelve machine learning models, including both deep learning and traditional approaches, were independently trained and evaluated. Accuracy, sensitivity, and specificity were calculated for each model. Finally, the best four top-performing models were further combined using a stacking strategy for final prediction.

**Results**

The study included good-prognosis females (average age: 29.55 ± 2.94 years) with an adequate number of Day 3 embryos (median: 9 [7, 11]). Supervised machine learning of labeled Raman spectra revealed distinct clusters for each group. The best-performing models were multilayer perceptron, artificial neural network, gated recurrent unit, and linear discriminant analysis. Using the stacking strategy, two samples were misclassified, and 33 were correctly predicted. Sensitivity for A, B, and C predictions was 0.92, 1.00, and 0.94, respectively. Specificity for A, B, and C predictions was 1.00, 0.93, and 1.00, respectively. The overall accuracy, sensitivity, and specificity were 0.94, 0.93, and 0.97, respectively.

**Conclusion:**

Our preliminary study suggests that machine learning combined with Raman spectra of spent Day 3 culture medium represents a promising non-invasive approach for embryo selection at the cleavage stage.

## 1 Introduction

Assisted reproductive technology (ART) is the most effective treatment for infertility. In ART, Day 3 embryos (cleavage stage) or Day 5–6 embryos (blastocyst stage) are most commonly used for transfer in reproductive centers worldwide. Due to the fact that many Day 3 embryos cannot progress to the blastocyst stage, blastocysts generally have better developmental potential than Day 3 embryos, as indicated by higher implantation and live birth rates (1, 2). Additionally, single blastocyst transfer effectively reduces the risk of multiple pregnancies (3–5). As a result, extended culture of embryos to the blastocyst stage is increasingly prevalent.

However, with the rise in blastocyst transfer cycles, concerns regarding potential drawbacks of blastocyst transfer have emerged. Blastocyst transfers have been linked to a higher risk of preterm delivery, large-for-gestational-age infants, monozygotic twins, and altered sex ratios compared to Day 3 embryo transfers (6). A recent study suggested that blastocyst transfer, not Day 3 embryo transfer, may be associated with shorter leukocyte telomere length in ART-conceived children, suggesting that blastocyst transfer could potentially accelerate aging in offspring (7). It is proposed that extended culture could introduce epigenetic changes that affect offspring health (7, 8). Since the first blastocyst transfer birth is only 33 years old, long-term safety concerns remain. This has led researchers to question, “Should we be promoting blastocyst-stage embryo transfer?” (6). Since extended culture is an effective form of natural selection, identifying high-potential Day 3 embryos capable of developing into good quality blastocysts without extended culture may help address this issue.

Currently, morphological scoring remains the most widely used method for assessing embryo quality, owing to its non-invasive, convenient, and relatively reliable properties. However, morphological scoring is less objective (9). Although Day 3 embryos with good morphology are more likely to form blastocysts compared to those with poor morphology, morphological scoring cannot reliably distinguish embryos that will develop into blastocysts from those that will not (10). Thus, new methods for assessing embryo quality are urgently needed.

Metabolic differences (uptake and secretion) between high-and low-quality embryos have been demonstrated, and researchers have worked to reveal these differences in spent culture media to predict developmental potential through metabolomic profiling (11, 12). Raman spectroscopy, a type of scattering spectroscopy, can detect extensive molecular information, including in liquids, with only a small sample volume (10 µL). This method is simple, non-invasive, and fast, making it well-suited for metabolomic profiling of spent human embryo culture media (13). Several studies have shown promising results in using Raman spectroscopy of spent culture media to identify high-quality embryos (14–17). Liang et al. reported that Raman spectroscopy could distinguish aneuploid from euploid embryos (18). A recent study showed that combining Raman spectroscopy of Day 3 high-quality embryo spent culture medium with deep learning can identify embryos with blastocyst development potential with an accuracy of 73.5% (19). However, in our IVF lab, the blastocyst formation rate from good-quality Day 3 embryos is already 70-73% (20), so this level of accuracy has limited clinical significance. Additionally, the developmental potential of clinically useful blastocysts varies significantly, with morphologically good blastocysts having substantially higher developmental potential than poor-quality ones (21). Thus, using Raman spectroscopy of Day 3 culture media to select morphologically good blastocysts is of greater importance.

In this study, we collected 172 spent culture media samples from Day 3 embryos including good and poor morphological score embryos, with known extended culture outcomes. Metabolic profiling of the culture media was measured by using Raman spectroscopy. Extended culture outcomes were categorized into three groups: morphologically good blastocysts (group A), morphologically non-good blastocysts (group B), and clinically non-useful embryos (group C), including those that failed to develop into or became unusable blastocysts. Machine learning was applied to correlate extended culture outcomes with Raman spectral features of the Day 3 media. Of the samples, 137 were used as a training dataset, and 35 as a prediction dataset. Predicted outcomes were then compared to actual extended culture results.

## 2 Materials and methods

### 2.1 Study design

**Figure 1:**
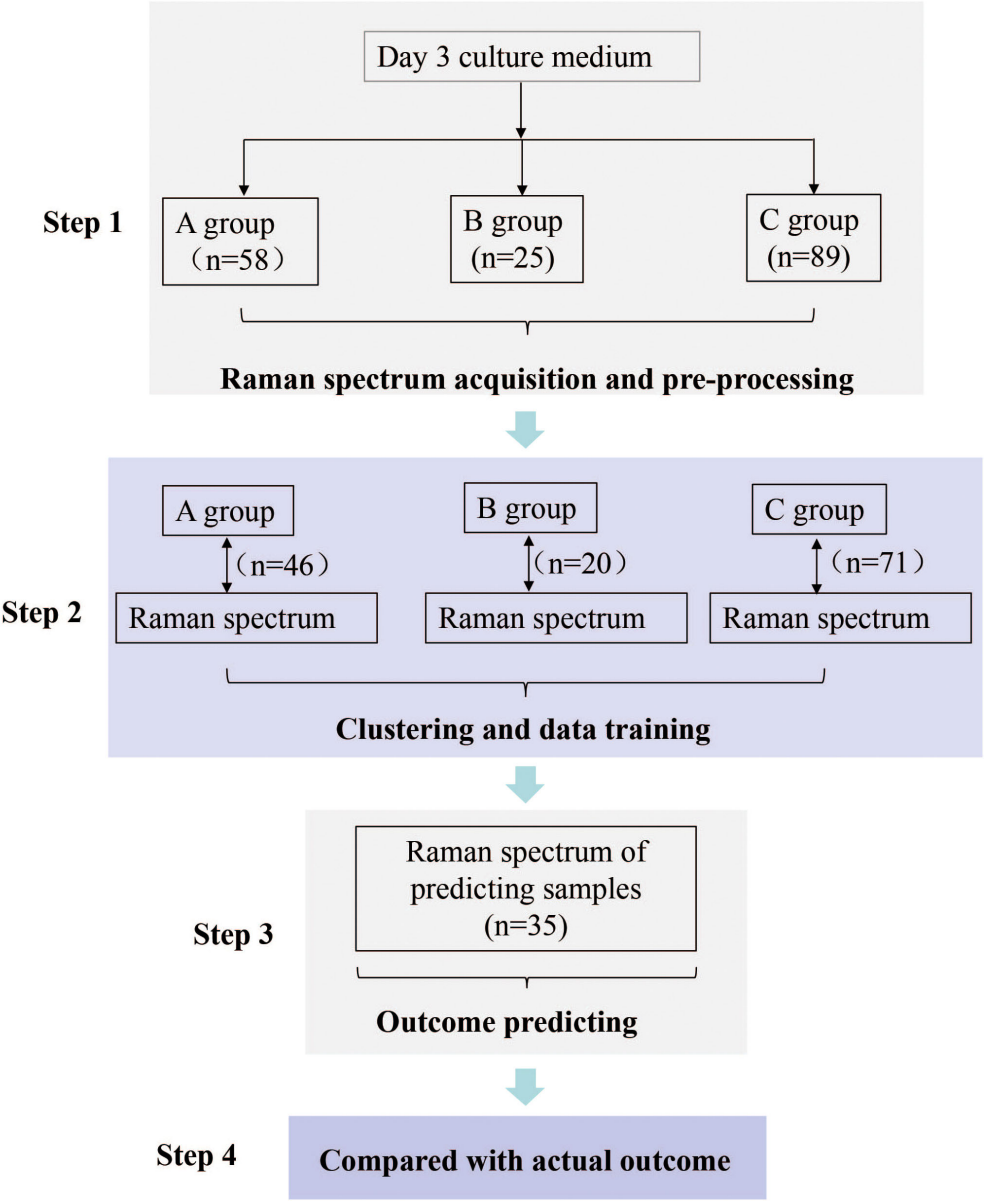
Flowchart of the Study. Step 1: Based on extended culture outcomes, spent Day 3 culture medium samples were categorized into three groups: A, B, and C. Raman spectra were collected and pro-processed. Step 2: Machine learning was applied to correlate Raman spectra from a randomly selected 80% of samples in each group with the known extended culture outcomes for clustering and data training. Step 3: Raman spectra from the remaining samples were used to make predictions. Step 4: Predicted result was compared with actual outcome. A: Morphologically good blastocyst; B: Morphologically non-good blastocyst; C: Clinically non-useful embryos.

Infertile couples who sought IVF-ET assistance for pregnancy at our reproductive center between February 2020 and February 2021 were included in this study. We selected a total of 172 culture medium samples from day 3 embryos that underwent extended culture. Based on the extended culture outcomes, all the samples were categorized into three groups: group A (n=58), group B (n=25), and group C (n=89). For each group, 80% of the samples were used as the training dataset, while the remaining samples were used as the prediction dataset. Details of the study design are presented in **Figure 1**.

### 2.2 Embryo culture and embryo score

After oocyte retrieval, oocytes were fertilized either by conventional *in vitro* fertilization or ICSI in IVF medium. The day of insemination was designated as day 0. Approximately 18 hours later, the zygotes were observed under a light microscope. Fertilization was assessed by the presence of pronuclei in the zygotes. Normal fertilization was identified by the presence of two pronuclei (one from the father and one from the mother), and zygotes were then cultured in G1 medium (Vitrolife, Sweden). On day 3, embryos were graded based on our previous methodology (10). According to blastomere count and size, fragmentation percentage, and other criteria, Day 3 embryos were classified into four groups (Grade I, II, III, and IV) in ascending order of developmental potential. Grade IV embryos were discarded due to a lack of further developmental potential. Day 3 embryos with developmental potential were transferred to G2 medium (Vitrolife, Sweden) for extended culture. On day 5 or day 6, embryos that reached the blastocyst stage were evaluated using a morphological scoring system based on blastocyst expansion, as well as the scores of the inner cell mass (ICM) and trophectoderm (TE), as previously described (10). Blastocyst with an expansion grade of 3 or higher and an ICM and TE score of either A or B were considered morphologically good embryos (group A). Blastocyst with an expansion grade of 3 or higher, a C score in either ICM or TE, and a corresponding A or B score in the other component (ICM or TE) were considered morphologically non-good embryos (group B). Both morphologically good and non-good blastocysts were clinically useful. Embryo that either failed to reach the blastocyst stage or had a C score in both ICM and TE were considered as clinically non-useful embryos (group C). The detailed morphological appearance of blastocyst belongs to group A, B and C was listed in **Supplementary Figure 1**.

### 2.3 Sample collection and treatment

A 25 µL drop of spent culture medium from Day 3 embryos was immediately collected when the embryo was transferred to G2-plus medium for extended culture. The medium was centrifuged at 1438 g for 10 minutes to separate the culture medium from the paraffin oil, which is commonly used to cover the culture drops in the IVF lab. The culture medium was then collected and stored at -80°C for later use.

### 2.4 Raman spectroscope detection and analysis

Ten microliters of collected medium was placed onto a tray and air-dried. Several sites on the air-dried medium were selected to acquire Raman spectra, with five spectra collected per site. A total of 30 to 40 Raman spectra for each sample was acquired for analysis. Raman spectroscopy detection and analysis were performed as previously described (22). A WITec alpha300 Raman microscope (WITec GmbH, Germany) equipped with a 532 nm laser was used in this study. A 100x objective (Epiplan-Neofluar, NA = 0.9, Zeiss) was used to focus the excitation light. The laser power was approximately 15 mW, and a grating with 600 g/mm was employed to disperse the Raman emission for wavelength recording. The acquisition time was set to 3–5 seconds, and the Raman spectral shift ranged from 300 cm^-1^ to 3400 cm^-1^.

### 2.5 Raw Raman spectra preprocessing

Raman spectral preprocessing was performed through a standardized multi-step pipeline to enhance data quality and comparability by using Labspec 6 (HORIBA, Japan). First, abnormal spectra exhibiting excessive noise or baseline distortion were excluded based on signal-to-noise ratio and spectral profile inspection. To eliminate sharp intensity spikes caused by cosmic rays, a dynamic filtering algorithm was applied with a filter size of 4 and a dynamic factor of 6. The spectra were then truncated to the wavenumber range of 300 to 3400 cm^-1^, and resampled at 1 cm^-1^ intervals to ensure uniform spectral resolution and alignment across all samples. Baseline correction was conducted using the Asymmetric Least Squares (AsLS) algorithm, which effectively removed broad background variations while preserving true Raman features. Subsequently, a Savitzky–Golay filter was employed (window length = 10, polynomial order = 3) to reduce high-frequency noise and smooth the spectral curves without distorting peak shapes. Finally, area normalization was applied by scaling the total intensity of each spectrum to a fixed value of 100, allowing for consistent comparison across different samples regardless of absolute signal strength. This preprocessing strategy ensured robust and reliable input for downstream statistical and machine learning analyses.

### 2.6 Classification of core peak intensity patterns among groups

The mean intensities of core peaks were compared across groups. Classifications were based on the value of mean intensities and the presence of statistically significant differences:

1. If the mean intensities follow the order A > B > C, A < B < C, B < A < C, or A < C < B, with statistically differences between each pair of groups, they are classified respectively as: A > B > C, A < B < C, B < A < C, or A < C < B.
2. If the mean intensity follows A ≈ B < C, with statistically significant differences between both A vs. C and B vs. C, but not between A and B, it is classified as: A ≈ B < C.
3. If the mean intensity follows A > B ≈ C, with significant differences between A vs. B and A vs. C, but not between B and C, the classification is: A > B ≈ C.
4. If the mean intensity follows A ≈ C > B, with significant differences between A vs. B and C vs. B, but not between A and C, it is classified as: A ≈ C > B.
5. If only one pair of groups shows a statistically significant difference in mean intensity, such as B > A or C > B, it is classified accordingly as: B > A or C > B.
6. If no statistically significant differences in mean intensity are observed among any of the groups, the classification is: A ≈ B ≈ C.

### 2.7 Visualization of Raman spectra data

Over 1,000 intensity values across positions ranging from 300 cm^-1^ to 3400 cm^-1^ were extracted from each individual spectrum, generating high-dimensional data. Machine learning algorithms can manage this vast amount of information by applying dimensionality reduction for visualization (23). In this study, both unsupervised and supervised algorithms were utilized. For the unsupervised approach, the t-distributed stochastic neighbor embedding (t-SNE) method, a nonlinear dimensionality reduction algorithm, was used due to its effectiveness in dimensionality reduction and clustering (24). For supervised analysis, Latent Dirichlet Allocation (LaDA) and Orthogonal Partial Least Squares Discriminant Analysis (OPLS-DA) were employed. LaDA and OPLS-DA are powerful dimensionality reduction tools, particularly useful in supervised learning contexts (25, 26). LDA identifies topic distributions that optimize prediction of the target variable, while OPLS-DA isolates components that maximize variance between classes, filtering out variance unrelated to class separation.

### 2.8 Algorithm models for data training and prediction

Eighty percent of samples from each group were randomly selected as the training dataset, and the remaining samples were designated as the prediction dataset. To enhance model training efficiency, the synthetic minority oversampling technique was applied to the training dataset (27). Raman spectra labeled with known extended culture outcomes were used for training, while unlabeled spectra were predicted using 12 algorithmic models, including multilayer perceptron (MLP) (28), artificial neural network (ANN) (29), gated recurrent unit (GRU) (30), gradient boosting (GB) (31), K-nearest neighbors (KNN) (32), random forest (RF) (33), linear support vector machine (LSVM) (34), linear discriminant analysis (LDA) (35), logistic regression (LR) (36), and quadratic discriminant analysis (QDA) (37), reduced support vector machines (RSVM) (38), and naïve Bayes (NB) (39). Among these, MLP, ANN, and GRU belong to deep learning models, while the others are traditional machine learning models. For traditional machine learning models, 5-fold cross-validation (k=5) was applied to optimize hyperparameters and evaluate model performance. Accuracy, sensitivity, and specificity were calculated for each model. The training and validation accuracy and loss, prediction results for spectra and samples, and ROC curves for predicting A, B, and C were presented for the best-performing model. Next, model stacking was applied to improve predictive performance. Model stacking is an ensemble technique that combines multiple models to increase accuracy, reduce over-fitting, and utilize diverse models (40). In stacking, different models are trained independently on the same dataset, and their predictions are used as inputs to a “meta-model” or “stacked model,” which learns to make a final prediction by leveraging the strengths of each model (41). The four best-performing models in this study were selected for stacking. Finally, predictions from multiple Raman spectra within a single sample were aggregated using a mode-based mechanism to make the final decision.

### 2.9 Software

All model analyses were conducted using Python 3.9. The libraries employed included, but were not limited to, SciPy for statistical analysis, scikit-learn for machine learning models, TensorFlow and PyTorch for deep learning models, and Matplotlib and Seaborn for data visualization.

## 3 Results

### 3.1 Patient characteristics and details of embryo culture

A total of 172 samples of spent Day 3 culture medium were collected from 78 couples undergoing IVF treatment (**Table 1**). The mean age of the women was 29.55 ± 2.94 years (**Table 1**). The primary infertility rate was 57.7%, and 85.9% of the women underwent an ovulation induction protocol with an agonist (**Table 1**). The median number of oocytes retrieved was 11.5 [IQR: 9.75, 15] (**Table 1**). Conventional *in vitro* fertilization was used in 87.2% of cases, and the median number of available Day 3 embryos was 9 [IQR: 7, 11] (**Table 1**). A total of 82 culture medium samples from Grade I and Grade II embryos were collected; of these, 38 embryos were in group A, 2 embryos in group B, and 42 embryos in group C (**Table 1**). Additionally, 90 culture medium samples from Grade III embryos were collected, of which 20 were in group A, 23 embryos in group B, and 47 embryos in group C (**Table 1**).

**Table 1:** Patient characteristics and details of embryo culture.

n	78
Age	29.55 ± 2.94
Primary infertility (%)	45 (57.7)
Ovulation induction protocols (%)	
Agonist	67(85.9)
Antagonist	9 (11.5)
Mild stimulation	2 (2.6)
Occytes retrieved	11.5 [9.75, 15]
IVF cycles (%)	68 (87.2)
Available day 3 embryos	9 [7, 11]
Grad I+II embryos (82)	
A	38
B	2
C	42
Grad III embryos (90)	
A	20
B	23
C	47

Data expressed as mean ± SD or count (percentage) or medium [15% percentile,75% percentile].

IVF: *In vitro* fertilization; A: morphologically good blastocyst; B: morphologically non-good blastocyst; C: clinically non-useful embryos.

### 3.2 Raman finger print and patterns of core peak intensity among groups

**Figure 2:**
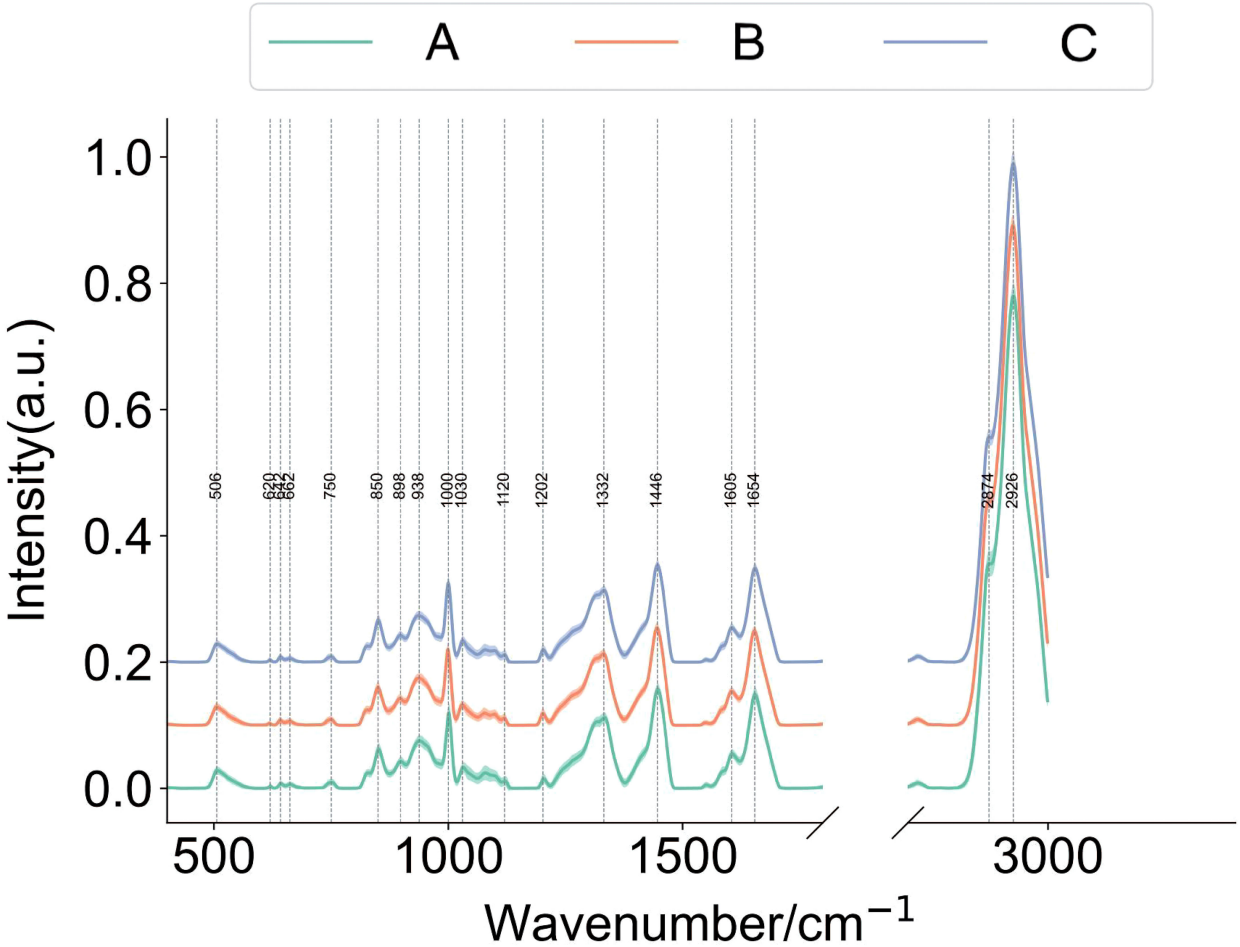
Raman spectra among groups. Overview of core peaks in Raman spectra from Day 3 spent culture medium for A, B, and C groups. A: Morphologically good blastocyst; B: Morphologically non-good blastocyst; C: Clinically non-useful embryos.

**Figure 3:**
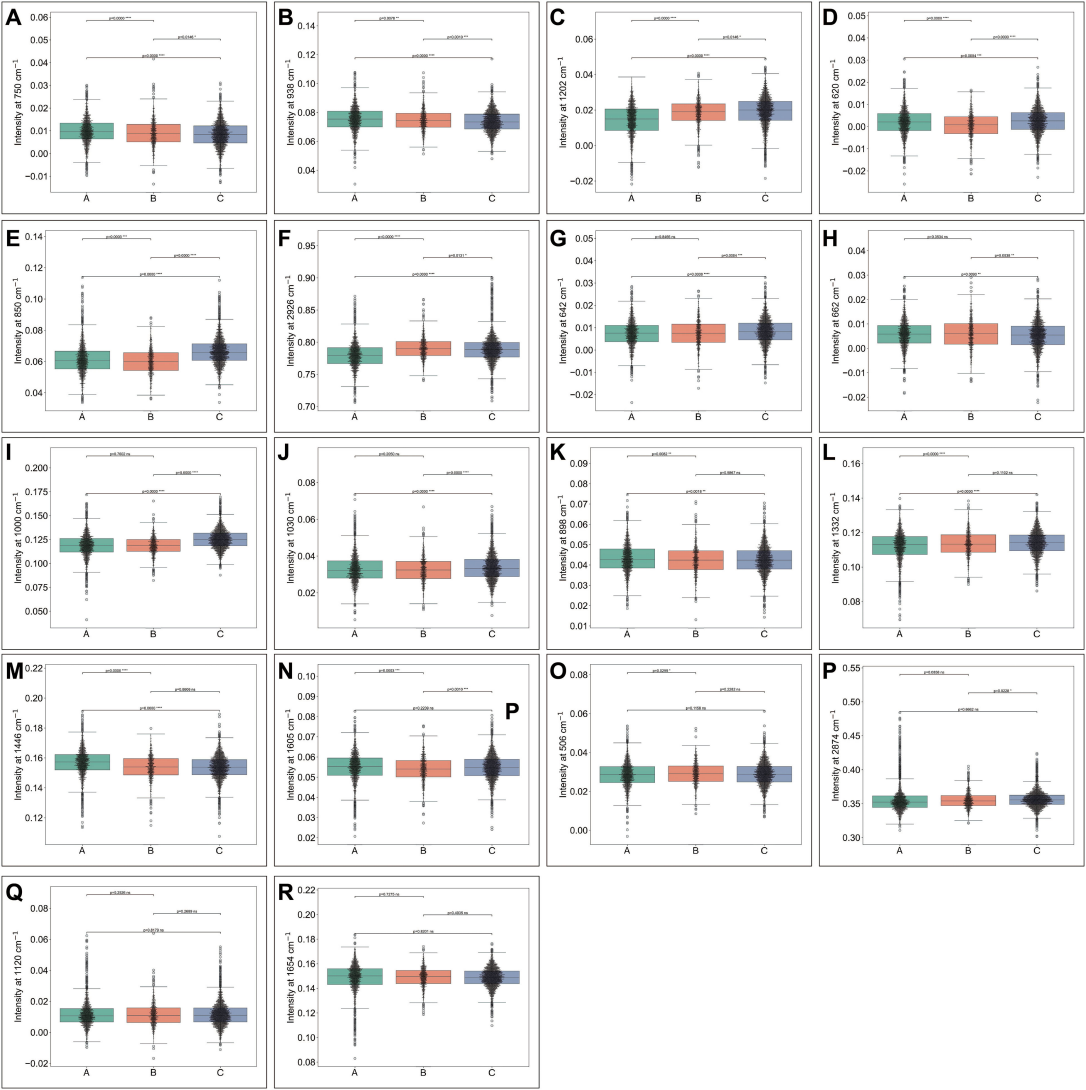
Core peak intensity patterns in Raman spectra among groups. The core peak intensity at various positions showed distinct intensity patterns across groups. At 750 cm^-1^ **(A)** and 938 cm^-1^ **(B)** showed: A > B > C. At 1202 cm^-1^ **(C)** showed: A < B < C. At 620 cm^-1^ **(D)** and 850 cm^-1^ **(E)** showed: B < A < C. At 2926 cm^-1^ **(F)** showed A < C< B. At 642 cm^-1^ **(G)**, 662 cm^-1^ **(H)**, 1000 cm^-1^ **(I)**, and 1030 cm^-1^ **(J)** showed:A ≈ B < **(C)** At 898 cm^-1^ **(K)**, 1332 cm^-1^ **(L)**, and 1446 cm^-1^ **(M)** showed: A > B ≈ C. At 1605 cm^-1^ **(N)** showed A ≈ C > B. At 506 cm^-1^ **(O)** showed: B > A. At 2874 cm^-1^ **(P)** showed C > B. At 1120 cm^-1^ **(Q)** and 1654 cm^-1^ **(R)**: A ≈ B ≈ C. *p < 0.05; **p < 0.01; ***p < 0.001. ns: no significant difference. A: Morphologically good blastocyst; B: Morphologically non-good blastocyst; C: Clinically non-useful embryos.

Each Raman spectrum in each group included 18 core peak positions: 506, 620, 642, 662, 750, 850, 938, 1000, 1030, 1120, 1202, 1332, 1446, 1605, 1654, 2874, and 2926 cm^-1^ (**Figure 2**). The statistical analysis of intensity of core peaks at different positions among three groups were shown in **Figures 3A–R**. Combining with the value of mean peak intensity and statistical significance, we summarized a total of ten patterns of peak intensity among groups, including A > B > C (**Figures 3A, B**), A < B < C (**Figure 3C**), B < A < C (**Figures 3D, E**), A <C < B (**Figure 3F**), A ≈ B < C (**Figures 3G–J**), A > B≈C (**Figures 3K–M**), A ≈ C > B (**Figure 3N**), B > A (**Figure 3O**), C > B (**Figure 3P**) and A ≈ B ≈ C (**Figures 3Q,R**). These results revealed distinct, complicated and subtle spectral differences among groups, and indicated that relaying on peak core intensity difference is not likely to effectively distinguish different outcomes.

### 3.3 Group-specific clustering of Raman spectra

**Figure 4:**
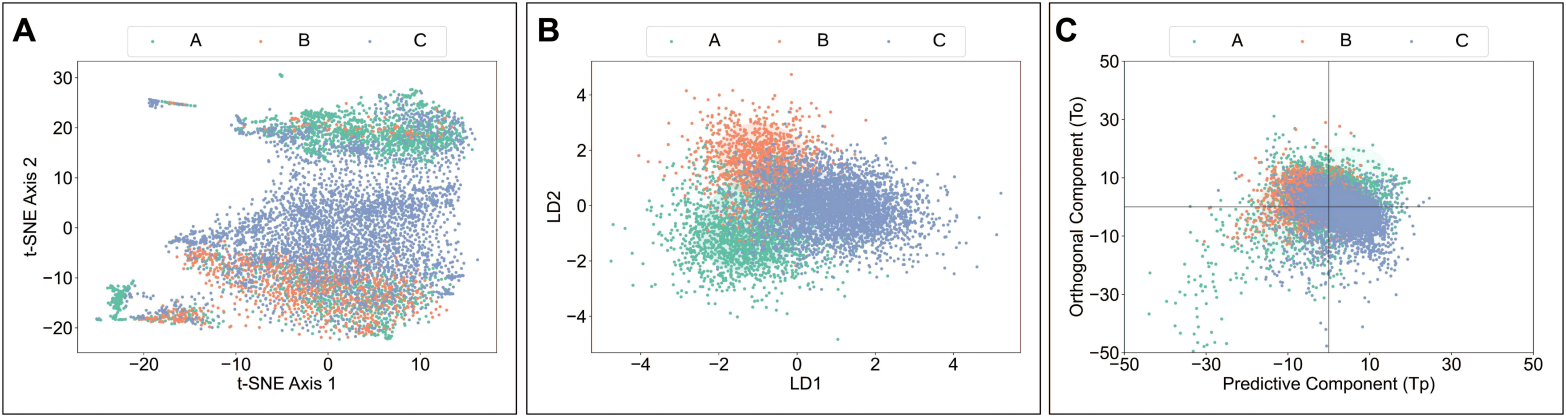
Group-specific clustering of Raman spectra. Dimensionality reduction techniques applied to Raman spectra for visualization among groups: **(A)** t-SNE, **(B)** LaDA, and **(C)** OPLS-DA. A: Morphologically good blastocyst; B: Morphologically non-good blastocyst; C: Clinically non-useful embryos; t-SNE, t-Distributed Stochastic Neighbor Embedding; LaDA, Latent Dirichlet Allocation; OPLS-DA, Orthogonal Partial Least Squares Discriminant Analysis.

Dimensionality reduction by unsupervised algorithms, t-SNE, revealed rough three clusters according to the groups (**Figure 4A**). However, many spectra from the A and B groups were intermixed with those from the C group (**Figure 4A**). We then applied two supervised algorithms for visualization: LaDA and OPLS-DA, which take into account both spectral features and sample labels. Compared to the t-SNE distribution, LaDA and OPLS-DA produced a more distinct clustering of spectra by group with less overlap (**Figures 4B, C**). These findings revealed group-specific differences in the Raman spectra of the culture medium.

### 3.4 MLP model had the best ability for predicting

**Figure 5:**
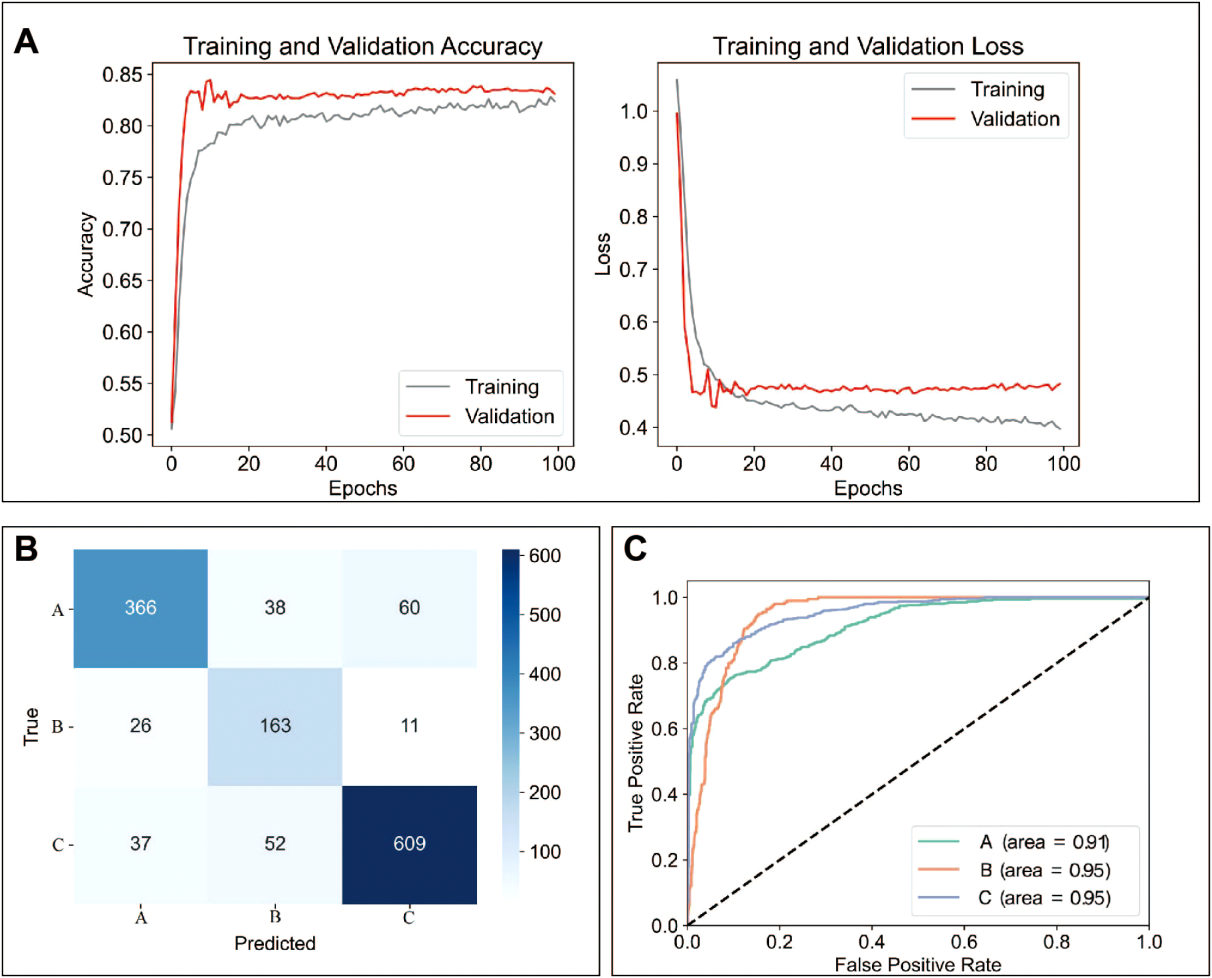
MLP model had the best predicting ability. **(A)** Validation accuracy and loss during data training using the MLP. **(B)** Comparison of actual and predicted extended culture outcomes of Raman spectra using the MLP model. **(C)** AUC curves for predicted extended culture outcomes of A, B, and C groups. A: Morphologically good blastocyst; B: Morphologically non-good blastocyst; C: Clinically non-useful embryos; MLP: multilayer perceptron model.

A total of 12 models were used for training and predicting. The accuracy, sensitivity, and specificity for each model were presented in **Table 2**. Among these, the MLP model had the best predictive performance (**Table 2**). Training and validation accuracy, as well as training and validation loss, reflect the model’s performance on the training and validation datasets (42). Our results showed that the MLP model performed well in both the training and prediction datasets (**Figure 5A**). The prediction results for Raman spectra in the test dataset using the MLP model were shown in **Figure 5B**. The sensitivity for predicting A was 0.79, for predicting B was 0.82, and for predicting C was 0.87, while the specificity for predicting A was 0.93, for predicting B was 0.92, and for predicting C was 0.89. The ROC curve for predicting A, B, and C was shown in **Figure 4C**. The AUC for A was 0.91, and for B and C, it was 0.95 (**Figure 5C**).

**Table 2:** The predicting efficiency of different models.

Rank	Model	Accuracy	Sensitivity	Specificity
1	MLP	0.84	0.83	0.92
2	GRU	0.81	0.76	0.89
3	ANN	0.81	0.76	0.9
4	LDA	0.78	0.75	0.88
5	QDA	0.77	0.72	0.86
6	GB	0.77	0.71	0.87
7	RF	0.77	0.7	0.86
8	NB	0.76	0.73	0.86
9	LR	0.76	0.69	0.85
10	LSVM	0.74	0.72	0.86
11	KNN	0.71	0.59	0.83
12	RSVM	0.53	0.35	0.68

The predicting efficiency for a total of 12 models from rank 1 to rank 12 with the data of predicting accuracy, sensitivity and specificity. MLP, multilayer perceptron; ANN, artificial neural network; GRU, gated recurrent unit; LDA, linear discriminant analysis; QDA, quadratic discriminant analysis.GB, gradient boosting; RF, random forest; NB, naïve Bayes; LR, logistic regression; LSVM, linear support vector machine; KNN, K-nearest neighbors; RSVM, Reduced support vector machines.

### 3.5 The stacking strategy accurately predicted the outcomes of extended embryo culture

**Figure 6:**
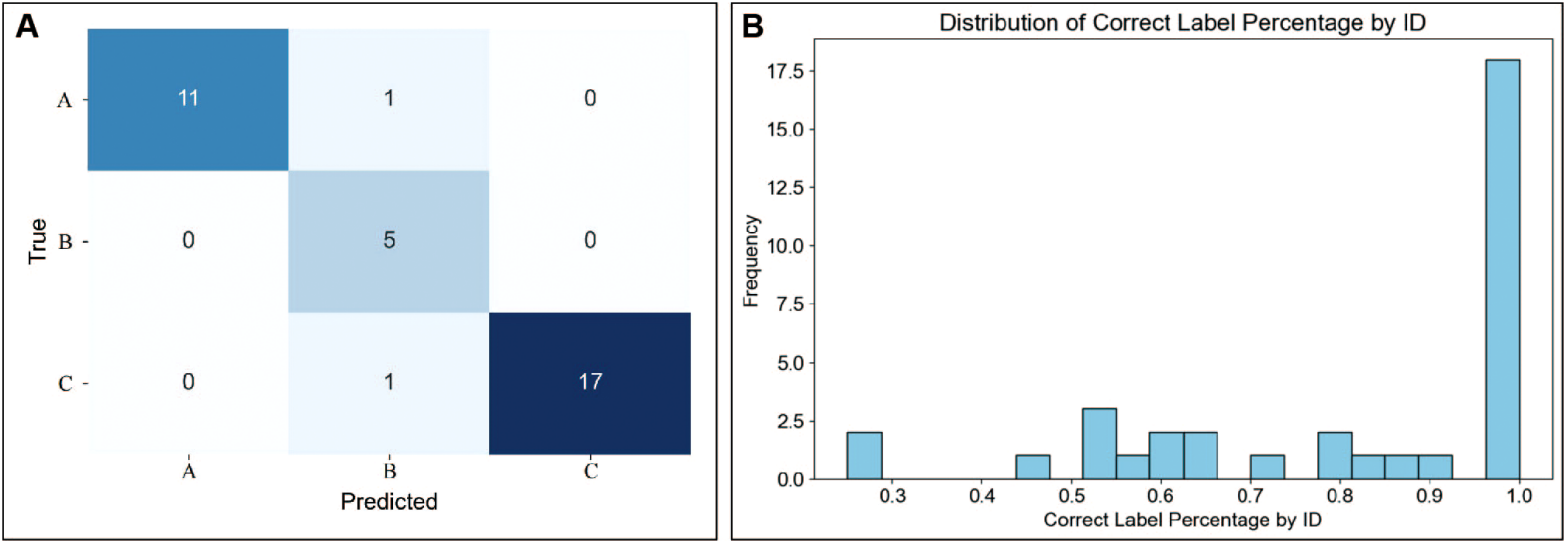
The stacking strategy accurately predicted the outcomes of extended embryo culture. **(A)** Comparison of actual and predicted extended culture outcomes for each sample using a model stacking strategy. **(B)** Distribution of samples showing the percentage of correctly predicted Raman spectra out of all Raman spectra for each sample. A: Morphologically good blastocyst; B: Morphologically non-good blastocyst; C: Clinically non-useful embryos.

To further improve prediction efficiency, the best four models (MLP, ANN, GRU, and LDAB) were stacked. The prediction results are presented in **Figure 6A**. The sensitivity for predicting A was 0.92, for predicting B was 1.0, and for predicting C was 0.94, while the specificity for predicting A was 1.0, for predicting B was 0.93, and for predicting C was 1.0. The overall accuracy was 0.94, the overall sensitivity was 0.93, and the overall specificity was 0.97. Additionally, over 91% of the samples had an accuracy rate of Raman spectra exceeding 50% (**Figure 6B**). For example, if each sample had 20 individual spectra, more than 91% of the samples have at least 10 spectra with correct prediction results.

## 4 Discussion

In the present study, machine learning was combined with Raman spectra of spent Day 3 culture medium to predict extended culture outcomes. Our preliminary results are promising, and indicated that machine learning analysis of Raman spectra from spent Day 3 culture medium can accurately predict the extended culture outcomes with high accuracy, sensitivity, and specificity. This study may provide a non-invasive, rapid, and effective tool for selecting good potential cleavage-stage embryos for transfer, potentially reducing the blastocyst transfer cycles.

The patients included in this study were couples with a good prognosis and a certain number of available Day 3 embryos. For these couples, selecting the best Day 3 embryos for transfer is particularly important and necessary. Therefore, the inclusion of these couples was appropriate for this study. Studies using Raman spectra of spent culture medium to predict embryo developmental potential have been reported since 2007 (14). Several studies classified spent culture medium into two groups: clinical pregnancy and non-pregnancy (14, 17, 43). This classification assumes that embryos leading to clinical pregnancies have good developmental potential. Although this concept is logical, it has inherent flaws. Both embryo and non-embryo factors contribute to female infertility, meaning that an embryo failing to implant does not necessarily indicate poor quality (44, 45). This grouping approach can introduce unwanted bias into the analysis. In the present study, we used extended culture outcomes for grouping, as they are relatively objective. This grouping ensures more consistent data within each group, which may contribute to the strong predictive ability shown in this study.

A previous study used machine learning in combination with Raman spectra of spent Day 3 culture medium to predict extended culture outcomes (19). The design of that study is similar to the present one. However, there are several major differences between the two. First, in that study, the extended culture outcomes were classified into two groups: useful blastocysts versus non-useful embryos. It is known that morphologically good blastocysts have much better developmental potential than morphologically non-good blastocysts (46), even though both morphologically good and non-good blastocysts are considered useful. In the present study, we classified the extended culture outcomes into three groups: morphologically good group, morphologically non-good group, and clinically non-useful group. This classification allows us to select the good-quality blastocyst from the useful blastocysts. Second, unlike that study, which only included spent culture medium from good-quality Day 3 embryos, the present study included spent culture medium from both good (Grade I+II, 82 cases) and poor (Grade III, 90 cases) Day 3 embryos. This design enhances the representativeness and robustness of the study. Notably, grouping was based solely on extended culture outcomes, without considering Day 3 embryo morphology. This highlights that machine learning combined with Raman spectra of spent Day 3 culture medium can independently predict extended culture outcomes, serving as a morphology-independent evaluation system. Finally, while the previous study reported a prediction accuracy of ~73%, this is comparable to the actual blastocyst formation rate (~70%) from good Day 3 embryos in our IVF laboratory (20), limiting its clinical significance. In contrast, our model demonstrates much higher predictive efficiency with meaningful clinical applicability.

Previous studies have identified differences in one or more substances in spent culture medium between embryos with good and poor developmental potential (15, 47). It is known that morphologically good, morphologically non-good, and clinically non-useful embryos show progressively decreased potential. However, only the intensity of core peaks in the Raman spectra at 750, 938, and 1202 cm^-1^ showed significant increases or decreases in value from A to B and further to C. No similar trends were observed at other peak positions. Due to the small differences and the varied and complex pattern of intensity values among the groups, it is impossible to make accurate predictions based on single or few differences. Instead, integrating all features (not limited to peak intensity values) is essential for effective prediction. Therefore, machine learning is required to process such large and complex data. In the present study, we found that deep learning models, such as MLP, ANN, and GRU, performed exceptionally well in training and prediction, demonstrating the advantages of deep learning in handling such tasks (48). The effectiveness of algorithm stacking for prediction has been demonstrated. In this study, the stacking strategy achieved higher prediction accuracy than any single algorithm. Thus, the stacking approach used in this study enhances the accuracy of predictions.

The present study is clinically significant. Our findings may offer an independent and non-invasive method for assessing day 3 embryo quality. Clearly, the current data indicate that machine learning combined with Raman spectra of spent Day 3 culture medium performs better in predicting embryo quality than traditional morphological scoring. Most importantly, this method allows for the selection of the good-quality embryos at the cleavage stage, potentially reducing the need for blastocyst transfer cycles. For instance, Day 3 embryos predicted to form morphologically good blastocysts based on the Raman spectra of the culture medium could be prioritized for transfer. The remaining Day 3 embryos predicted to form morphologically non-good or clinically non-useful blastocysts could undergo further culture. Currently, processing a batch of 20 samples, from collection to result release, takes approximately 3.5 hours using a single Raman spectrometer. Throughput can be further increased with multiple instruments. Therefore, this technique holds potential for clinical translation.

There are several limitations in the present study. First, the number of spent culture medium samples was limited. A larger sample size will be collected and analyzed to improve the predicting model in future studies. Second, it is unclear whether the training and prediction models established in one IVF laboratory are suitable for use in other IVF labs. This needs to be further validated across multiple IVF centers. Third, the findings of this study show promise for prediction, but a randomized clinical trial is needed to assess whether the strategy of Raman spectra of spent Day 3 culture medium-guided embryo transfer improves clinical outcomes compared to traditional morphological scoring. Finally, embryo developmental speed (blastocysts form on day 5 or 6), which has been linked to embryo developmental potential (49), was not considered in the present study. This will be considered in the future study.

## 5 Conclusion

In summary, the present study indicates that machine learning combined with Raman spectroscopy of day 3 spent culture medium holds the potential to predict extended embryo culture outcomes with high accuracy, sensitivity, and specificity. This non-invasive approach offers a promising strategy for embryo selection at the cleavage stage, potentially enabling the benefits of extended culture while mitigating the risks associated with blastocyst transfer. However, larger datasets and further studies are needed to validate the clinical feasibility and reliability of this method for routine embryo selection.

## Data Availability

The original contributions presented in the study are included in the article/**Supplementary Material**. Further inquiries can be directed to the corresponding authors.
