# Supplementary material for: Embryo selection at the cleavage stage using Raman spectroscopy of day 3 culture medium and machine learning: a preliminary study
Source: Front Endocrinol (Lausanne). 2025 Sep 15;16:1608318. doi: 10.3389/fendo.2025.1608318 (PMC12477197; doi:10.3389/fendo.2025.1608318)
Supplement: Supplementary file 1 [file Image1.pdf]

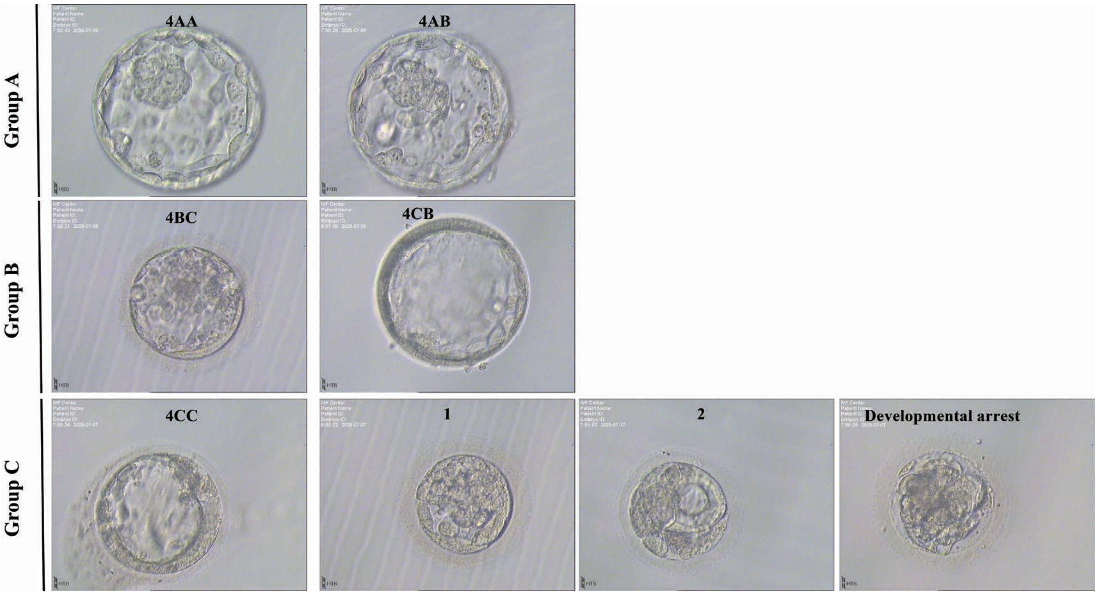

Supplementary Figure 1. Morphological appearance of blastocysts in Group A, Group B, and Group C. Group A included 4AA and 4AB blastocysts, which were considered morphologically good-quality blastocysts. Group B included 4BC blastocysts, classified as morphologically non-good-quality blastocysts. Group C included 4CC, early-stage (stage 1 and 2) blastocysts, and developmentally arrested embryos, which were considered clinically non-usable.
